# Effects of Different Nutrient Management Regimes on Rice Yield and Nitrogen Uptake and Use Efficiency

**DOI:** 10.3390/plants15101456

**Published:** 2026-05-10

**Authors:** Quanshi Feng, Gang Wu, Jiabao Wang, Qi Miao, Manman Yuan, Chuang Liu, Pingping Wu, Linsheng Yang, Zhili Sun, Chenshun Wang, Hong Wang, Yixiang Sun

**Affiliations:** 1 College of Resources and Environment, Anhui Science and Technology University, Fengyang 233100, China; 19565980865@163.com; 2 Key Laboratory of Nutrient Cycling and Arable Land Conservation of Anhui Province, Soil and Fertilizer Research Institute, Anhui Academy of Agricultural Sciences, Hefei 230031, China; gangw1987@163.com (G.W.); wangjiabao19900804@163.com (J.W.); miaoqibody@163.com (Q.M.); mmyuan09@163.com (M.Y.); pingpingwu1982@126.com (P.W.); zhilisun@ahstu.edu.cn (Z.S.); 15656252883@163.com (C.W.); 3 Rice Research Institute, Anhui Academy of Agricultural Sciences, Hefei 230031, China; yangls20160601@163.com

**Keywords:** slow/controlled-release fertilizer, nitrogen use efficiency, nitrogen uptake, rice yield

## Abstract

(1) Background: We investigated the effects of nutrient levels on rice yield and nitrogen uptake, with the aim of improving rice yield and nitrogen use efficiency. (2) Methods: A 3-year field experiment was conducted using the rice variety *Changliangyou Fuxiangzhan*, with six treatments: no nitrogen application (CK), conventional fertilization (FP), single basal application of 60-day slow-release urea (CRU1), single basal application of urea combined with 40-day and 90-day slow-release urea (CRU2), partial substitution of chemical fertilizer with bio-organic manure (FPM), and conventional fertilization combined with straw return (FPS). (3) Results: Different nutrient management regimes significantly affected rice yield and nitrogen uptake and use, as well as soil nitrogen content. CRU2 achieved the highest performance across most indicators, with grain yield averaging 9.6% higher than that of FP and 36.4% higher than that of CK, alongside consistently greater effective panicle numbers. It also significantly enhanced nitrogen uptake, with higher grain and straw N accumulation, and showed the best nitrogen use efficiencies. Soil responses varied by treatment: FPS and FPM increased total nitrogen, while CRU2 and CRU1 had the highest inorganic nitrogen, and microbial biomass nitrogen peaked under FPM, CRU2, and FPS. Despite these benefits, CRU2 showed the largest negative nitrogen balance, averaging −33.0 kg ha^−1^ over 3 years. (4) Conclusions: The CRU2 treatment achieved efficient synchronization between nitrogen supply and demand, with the highest yield, nitrogen uptake, and soil nitrogen levels.

## 1. Introduction

Rice is the staple food for half of the Chinese population, rendering its stable and high-yield production a cornerstone of national food security [1]. Nitrogen (N) is the primary nutrient limiting rice yield, making scientific N management pivotal for harmonizing high productivity with environmental stewardship [2]. However, current agricultural practices are often characterized by excessive N fertilization, leading to declining N use efficiency (NUE). This over-application of N leads to a series of environmental consequences, including groundwater contamination, aquatic eutrophication, and exacerbated greenhouse gas emissions [3,4]. Thus, identifying an optimal nutrient management regime that achieves spatial and temporal synchrony between N supply and crop demand is crucial for enhancing grain yield and NUE while mitigating ecological risks.

Studies have shown that partially substituting chemical fertilizer with bio-organic manure can increase rice yield and efficiency while improving soil physicochemical properties [5]. Straw return combined with chemical fertilizer significantly enhances soil nutrient levels, regulates soil pH, and effectively controls non-point source pollution [6]. As a new type of fertilizer, slow-release nitrogen fertilizer (SRF) regulates N release rates through physical or chemical means, achieving synchronization between N supply and crop uptake to a certain extent, reducing N losses, and improving fertilizer use efficiency [7]. Previous studies have demonstrated that co-applying slow-release fertilizers with conventional urea can effectively reduce ammonia volatilization and N leaching, significantly improve N use efficiency, and achieve stable yield increases [8,9]. A three-year field study found that a single basal application of slow-release urea increased rice yield and N use efficiency in cold regions while reducing N residue in black soil [10]. To date, several types of slow-release N fertilizers have been developed, including sulfur-coated urea, polyurethane-coated urea, and stabilized urea containing urease/nitrification inhibitors [11]. Sulfur-coated urea controls N release through a sulfur coating; however, its release rate is easily affected by soil microbial activity [12]. Urea containing urease inhibitors reduces ammonia volatilization mainly by delaying urea hydrolysis and has a relatively low cost. However, when used alone, its nutrient release control is unstable, and its capacity to regulate N supply throughout the entire growth period is limited [13]. In contrast, polyurethane-coated urea exhibits good nutrient controllability, achieves a relatively stable nutrient release curve, and optimally matches nutrient release with crop N demand [14].

However, most studies in this field have focused on the effects of combining a single type of slow-release fertilizer with conventional urea [10,11], largely neglecting systematic research on the combined application of different release-period products using the same coating material and its impact on rice yield formation, nitrogen uptake and utilization, and soil nitrogen pool dynamics. Rice N uptake exhibits distinct stage-specific characteristics: a substantial and concentrated demand during tillering, a gradual increase from jointing to booting, and a sustained requirement during grain filling to ensure plumpness [15,16]. A slow-release fertilizer with a single, fixed release pattern is therefore limited in its ability to perfectly synchronize with this dynamic N requirement across the entire growing season, often leading to either insufficient N supply during peak-demand periods or surplus N in the later stages [17]. Therefore, designing a N supply strategy that strategically combines SRFs with different release durations to achieve a high-fidelity match with the rice N uptake curve represents a critical frontier in nutrient management research.

Based on this, a multi-year field experiment was conducted to compare and analyze the effects of different nutrient management regimes on rice yield, yield components, nitrogen uptake and utilization efficiency, and soil nitrogen content. This study aimed to (1) evaluate the potential of combining polyurethane-coated urea with different release durations and fast-acting urea to increase yield and enhance nitrogen efficiency; (2) elucidate the mechanism by which blended slow-release nitrogen fertilizers regulate nitrogen supply to match the nitrogen demand patterns of rice; and (3) identify the optimal nutrient management regime for single-season rice production in the Jianghuai region.

## 2. Results

### 2.1. Grain Yield and Yield Components

#### 2.1.1. Grain Yield

**Figure 1:**
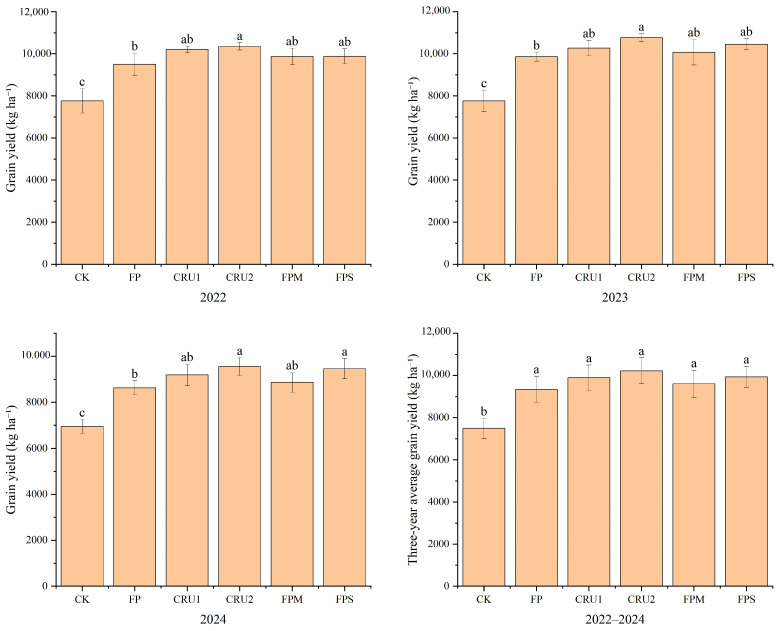
Grain yield of rice under different nutrient management regimes: annual grain yield, and three-year average grain yield. Vertical bars represent standard deviations (*n* = 3). Different lowercase letters above bars indicate significant differences among treatments at *p* < 0.05. CK, no nitrogen; FP, conventional farmer practice; CRU1, single basal application of 60-day slow-release urea; CRU2, single basal application of blended available urea with 40-day and 90-day slow-release urea (5:3:2 ratio); FPM, partial substitution of chemical fertilizer with bio-organic manure; FPS, conventional practice coupled with straw return.

The three-year field trial results demonstrated that different nutrient management regimes significantly affected rice yield. As shown in Figure 1, in 2022, compared with conventional fertilization (FP), CRU2 increased yield by 8.96%, showing a significant difference (*p* < 0.05). Compared with CK, all nitrogen application treatments significantly increased yield by 27.3–33.4%.

The yield trends in 2023 and 2024 were generally consistent. In 2023, compared with FP, CRU2 increased yield by 9.13%, showing a significant difference (*p* < 0.05). In 2024, compared with FP, CRU2 and FPS increased yield by 10.71% and 9.70%, respectively, both showing significant differences (*p* < 0.05). Compared with CK, all nitrogen application treatments resulted in significantly increased yields, namely by 27.1–38.7% in 2023 and by 24.2–37.4% in 2024.

The 3-year average yield was highest under CRU2 treatment, which was 9.6% higher than that under FP (*p* = 0.085) and 36.4% higher than that in the CK (*p* < 0.05). FPS and CRU1 treatments followed, with yields 6.5% and 6.0% higher than that in FP and 32.6% and 32.0% higher than that in CK, respectively. The yield in the FPM treatment was 3.0% higher than that in FP and 28.2% higher than that in CK. Regarding yield stability, the coefficient of variation for CRU2 was 6.01%, whereas FPS had the smallest coefficient of variation at 5.00%, both showing good yield stability. FP treatment resulted in the largest coefficient of variation at 6.79%, with obvious inter-annual fluctuations.

#### 2.1.2. Yield Components

Regarding yield components (Table 1), the effective panicle number across all treatments from 2022 to 2024 followed the order CRU2 > CRU1 > FPS > FPM > FP > CK. The effective panicle number under CRU2 was significantly higher than that under FP and CK across all years, with increases of 11.7%, 7.9%, and 11.0% compared with FP, and 30.0%, 29.5%, and 30.0% compared with CK, respectively.

No significant differences in spikelet number per panicle were observed among the N application treatments, but all N application treatments showed significantly higher values than the unfertilized control. CRU2 consistently maintained the highest values across all years. The spikelet number per panicle under CRU2 was 12.8%, 11.5%, and 15.1% higher than that under CK across the three years, with significant differences, but the increases compared with FP were small.

For seed setting rate, CRU2 showed significantly higher values than CK in 2022 and 2023, while no significant differences were observed among the other treatments. In 2024, no significant differences were observed among treatments. Thousand-grain weight and harvest index showed no significant differences among treatments from 2022 to 2024, mainly affected by the year.

Analysis of variance indicated that year significantly affected effective panicle number, seed setting rate, thousand-grain weight, and harvest index. Fertilization regime significantly affected spikelet number per panicle, seed setting rate, and effective panicle number. The interaction between year and treatment only significantly affected effective panicle number.

### 2.2. Nitrogen Uptake and Accumulation

**Figure 2:**
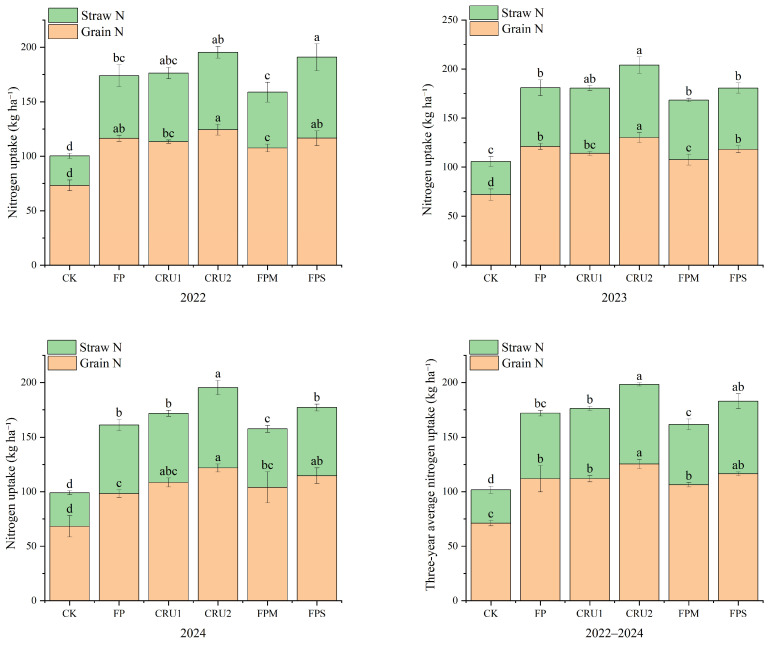
Nitrogen uptake in rice under different nutrient management regimes: grain N uptake, and straw N uptake. Vertical bars represent standard deviations (*n* = 3). Different lowercase letters above bars indicate significant differences among treatments at *p* < 0.05 within each year. Treatment abbreviations are defined in Figure 1.

Different nutrient management regimes significantly affected rice N uptake (Figure 2). In 2022, compared with FP, grain N uptake under FPM decreased by 7.6%, showing a significant difference (*p* < 0.05). In 2023, compared with FP, CRU2 increased grain N uptake by 7.5% (*p* < 0.05), while FPM decreased it by 11.1% (*p* < 0.05). Compared with CK, all N application treatments significantly increased grain N uptake by 46.9–81.2% in 2022–2023. In 2024, compared with FP, CRU2 and FPS increased grain N uptake by 24.0% and 16.8%, respectively, both showing significant differences (*p* < 0.05). Compared with CK, all N application treatments significantly increased grain N uptake by 43.8–78.3%.

For straw N uptake, in 2022, compared with FP, FPS increased it by 29.4%, showing a significant difference (*p* < 0.05). In 2023, CRU2 increased it by 23.4% (*p* < 0.05). Compared with CK, all N application treatments significantly increased straw N uptake by 76.3–174.5% in 2022–2023. In 2024, compared with FP, CRU2 increased straw N uptake by 17.2% (*p* < 0.05), while FPM decreased it by 14.6% (*p* < 0.05). Compared with CK, all N application treatments significantly increased straw N uptake by 74.2–139.1%.

On a 3-year average basis, grain N uptake followed the order CRU2 > FPS > CRU1 > FP > FPM > CK. CRU2 had the highest 3-year average grain N uptake at 125.46 kg ha^−1^, which was 12.1% higher than that of FP and 76.3% higher than that of CK, with significant differences (*p* < 0.05).

Straw N uptake on a 3-year average basis followed the order CRU2 > FPS > CRU1 > FP > FPM > CK. CRU2 had the highest 3-year average straw N uptake at 72.84 kg ha^−1^, which was 21.4% higher than that of FP and 138.0% higher than that of CK, with significant differences (*p* < 0.05).

### 2.3. Soil Nitrogen Dynamics

#### 2.3.1. Soil Total Nitrogen

**Figure 3:**
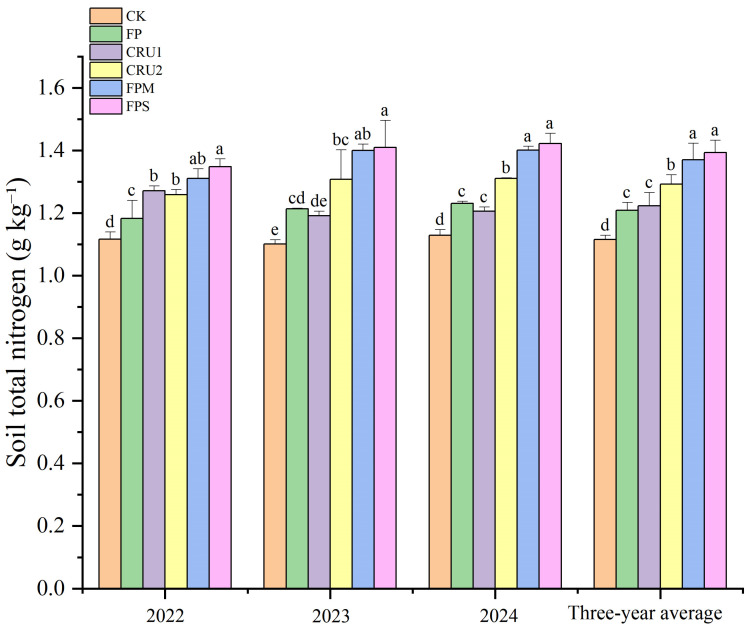
Soil total nitrogen content under different nutrient management regimes. Vertical bars represent standard deviations (*n* = 3). Different lowercase letters above bars indicate significant differences among treatments at *p* < 0.05 within each year. Treatment abbreviations are defined in Figure 1.

From 2022 to 2024, different fertilization regimes significantly affected soil total N content (Figure 3). In 2022, soil total N followed the order FPS > FPM > CRU1 > CRU2 > FP > CK. Compared with FP, FPS, FPM, CRU1, and CRU2 increased total N by 14.0%, 10.8%, 7.5%, and 6.4%, respectively, with FPS, FPM, and CRU1 showing significant differences (*p* < 0.05). Compared with CK, all N application treatments significantly increased total N by 6.0–20.8%. In 2023, compared with FP, FPS and FPM increased total N by 16.2% and 15.4%, respectively, both showing significant differences (*p* < 0.05). In 2024, compared with FP, FPS, FPM, and CRU2 increased total N by 15.5%, 13.8%, and 6.5%, respectively, with FPS and FPM showing significant differences (*p* < 0.05). Compared with CK, all N application treatments significantly increased total N by 6.8–28.0% in 2023–2024.

On a 3-year average basis, total N followed the order FPS > FPM > CRU2 > CRU1 > FP > CK. FPS and FPM had the highest 3-year average total N values, with increases of 15.2% and 13.4% compared with FP, respectively (*p* < 0.05). CRU2 showed a 6.7% increase compared with FP (*p* < 0.05).

#### 2.3.2. Soil Ammonium Nitrogen

**Figure 4:**
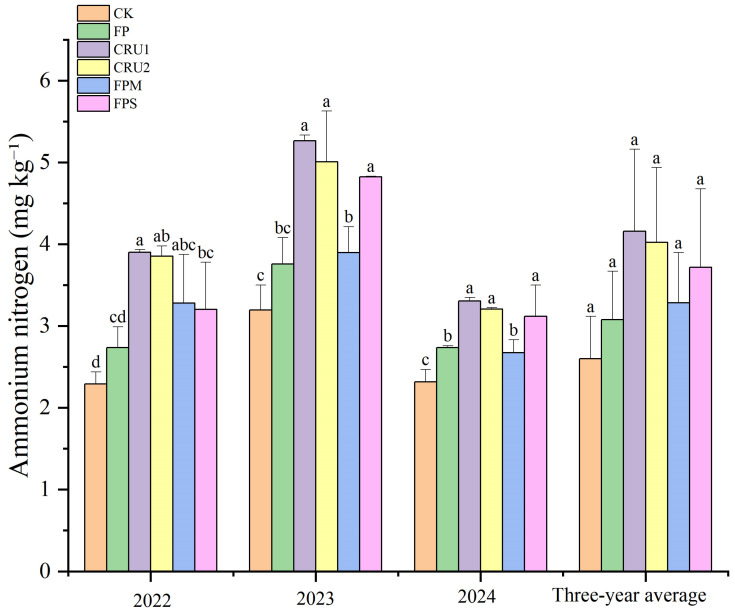
Soil ammonium nitrogen content under different nutrient management regimes. Vertical bars represent standard deviations (*n* = 3). Different lowercase letters above bars indicate significant differences among treatments at *p* < 0.05 within each year.

In 2022, compared with FP, CRU1 and CRU2 increased soil ammonium N content by 42.6% and 40.9%, respectively, both showing significant differences (*p* < 0.05) (Figure 4). Compared with CK, all N application treatments significantly increased ammonium N content by 19.5–70.4%. In 2023, compared with FP, CRU1, CRU2, and FPS increased ammonium N content by 40.0%, 33.2%, and 28.4%, respectively, all showing significant differences (*p* < 0.05). Compared with CK, all N application treatments significantly increased ammonium N content by 17.6–64.7%. In 2024, compared with FP, CRU1, CRU2, and FPS increased ammonium N content by 20.8%, 17.1%, and 14.0%, respectively, all showing significant differences (*p* < 0.05). Compared with CK, all N application treatments significantly increased ammonium N content by 15.4–42.7%.

On a 3-year average basis, ammonium N values followed the order CRU1 > CRU2 > FPS > FPM > FP > CK. CRU1 and CRU2 showed higher ammonium N values than FP, with increases of 35.1% and 30.6%, respectively, but these differences were not significant.

#### 2.3.3. Soil Nitrate Nitrogen

**Figure 5:**
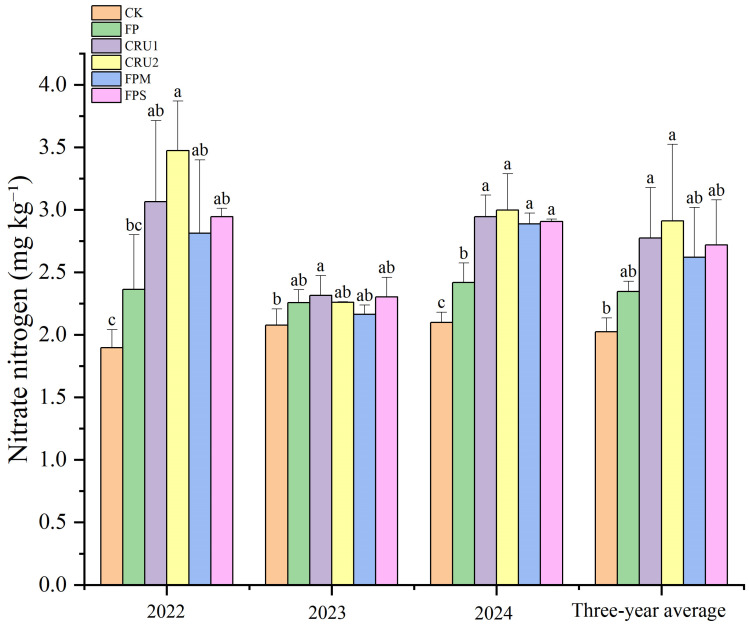
Soil nitrate nitrogen content under different nutrient management regimes. Vertical bars represent standard deviations (*n* = 3). Different lowercase letters above bars indicate significant differences among treatments at *p* < 0.05 within each year.

In 2022, compared with FP, CRU2 increased soil nitrate N content by 47.1%, showing a significant difference (*p* < 0.05) (Figure 5). Compared with CK, all N application treatments except FP significantly increased nitrate N content by 24.5–83.1%.

In 2023, compared with FP, no significant differences were observed among the N application treatments. Compared with CK, all N application treatments significantly increased nitrate N content by 4.2–11.5%.

In 2024, nitrate N values followed the order CRU2 > CRU1 > FPS > FPM > FP > CK. Compared with FP, CRU2, CRU1, FPS, and FPM increased nitrate N content by 23.9%, 21.7%, 20.2%, and 19.3%, respectively, all showing significant differences (*p* < 0.05). Compared with CK, all N application treatments significantly increased nitrate N content by 15.2–42.8%.

On a 3-year average basis, nitrate N values followed the order CRU2 > CRU1 > FPS > FPM > FP > CK. CRU2 and CRU1 showed higher nitrate N values than FP, with increases of 24.1% and 18.4%, respectively, but these differences were not significant.

#### 2.3.4. Soil Microbial Biomass Nitrogen

**Figure 6:**
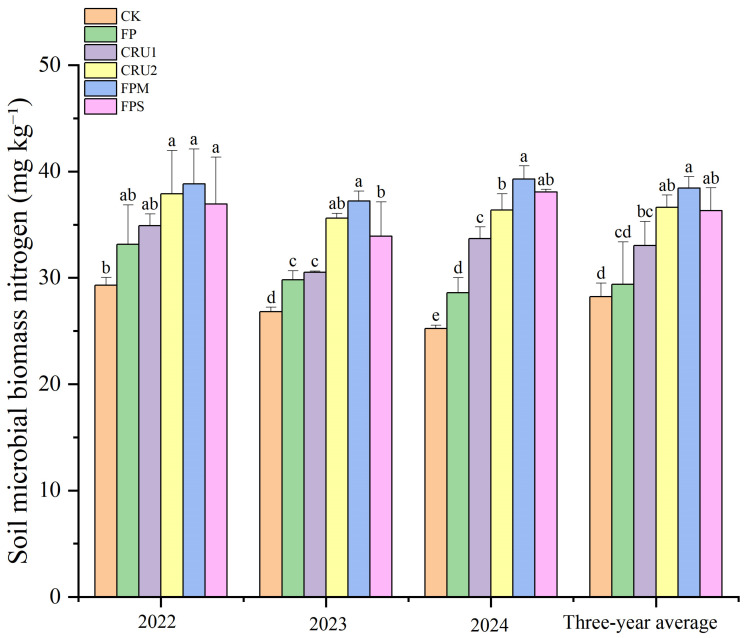
Soil microbial biomass nitrogen content under different nutrient management regimes. Vertical bars represent standard deviations (*n* = 3). Different lowercase letters above bars indicate significant differences among treatments at *p* < 0.05 within each year.

Regarding soil microbial biomass N, the 3-year trend was generally consistent. The 3-year average followed the order FPM > CRU2 > FPS > CRU1 > FP > CK (Figure 6). Compared with FP, FPM, CRU2, and FPS, they showed increases of 30.8%, 24.6%, and 23.5%, respectively, all showing significant differences (*p* < 0.05). Compared with CK, all N application treatments significantly increased microbial biomass N by 4.1–36.2%.

### 2.4. Nitrogen Use Efficiency

**Figure 7:**
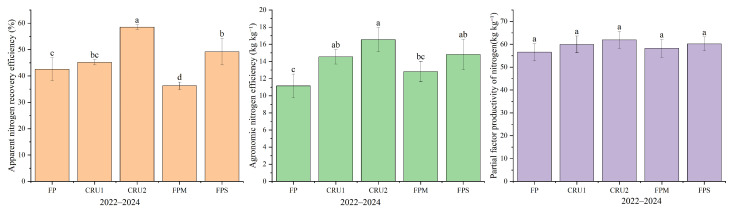
Three-year average nitrogen use efficiency under different nutrient management regimes: apparent nitrogen recovery efficiency (NRE, %), agronomic nitrogen efficiency (NAE, kg kg^−1^), and nitrogen partial factor productivity (PFPN, kg kg^−1^). Vertical bars represent standard deviations (*n* = 3). The nitrogen use efficiency values were calculated as three-year averages, excluding straw-derived nitrogen but including nitrogen from bio-organic manure. Different lowercase letters above bars indicate significant differences among treatments at *p* < 0.05. Treatment abbreviations are defined in Figure 1.

The effects of different nutrient management regimes on rice N use efficiency were generally consistent from 2022 to 2024, as analyzed using 3-year average values. As shown in Figure 7, the apparent N recovery efficiency (NRE) followed the order CRU2 > FPS > CRU1 > FP > FPM. CRU2 had the highest NRE at 58.52%, which was 15.98 percentage points higher than that of FP (*p* < 0.05) and 22.24 percentage points higher than that of FPM (*p* < 0.05). FPS had an NRE of 49.18%, which was 6.64 percentage points higher than that of FP, showing a significant difference (*p* < 0.05).

The agronomic N efficiency (NAE) followed the order CRU2 > FPS > CRU1 > FPM > FP. CRU2 showed an NAE value 5.40 kg kg^−1^ higher than that of FP (*p* < 0.05) and 3.73 kg kg^−1^ higher than that of FPM (*p* < 0.05). FPS and CRU1 had NAE values of 14.81 and 14.54 kg kg^−1^, respectively, with increases of 3.66 and 3.39 kg kg^−1^ compared with FP, both showing significant differences (*p* < 0.05). Nitrogen partial factor productivity (PFPN) was highest under CRU2, but no significant differences were observed among treatments.

These results indicate that CRU2 performed best in improving NRE, NAE, and PFPN, although FPS and CRU1 also showed certain promoting effects. In contrast, the performance of FPM was relatively poor in terms of NRE.

### 2.5. Nitrogen Balance

As shown in Table 2, the CRU2 treatment consistently exhibited the highest N output across all experimental years, with a 3-year average of 198.0 kg ha^−1^ (*p* < 0.05). This represented a substantial increase of 26.3 kg ha^−1^ (15.3%) compared to the conventional farmer practice (FP).

With the exception of the unfertilized control (CK), the CRU2 treatment showed the largest negative apparent N balance, averaging −33.0 kg ha^−1^ over the 3-year study period. This indicates that N uptake by the crop substantially exceeded N input, reflecting the highest N use efficiency among all fertilized treatments.

The FPS treatment achieved a N output of 191 kg ha^−1^ in 2022, which was statistically comparable to that of CRU2, with a corresponding apparent N balance of 2 kg ha^−1^. However, its performance declined in subsequent years, with the N output decreasing to 177–181 kg ha^−1^ in 2023 and 2024, suggesting limited stability in maintaining high N output over time.

The unfertilized control (CK) consistently recorded the lowest N output throughout the experiment, with a 3-year average of only 101.7 kg ha^−1^. As expected, it exhibited the largest negative apparent N balance, averaging −101.7 kg ha^−1^.

The FP, CRU1, and FPM treatments showed intermediate N output levels, ranging from 158 to 181 kg ha^−1^, with apparent N balances fluctuating between −16 and +7 kg ha^−1^. Notably, the FPM treatment exhibited positive N balances of +6 to +7 kg ha^−1^ in 2022 and 2024, indicating a lower N uptake efficiency compared to those of the other fertilized treatments.

### 2.6. Correlation Analysis

**Figure 8:**
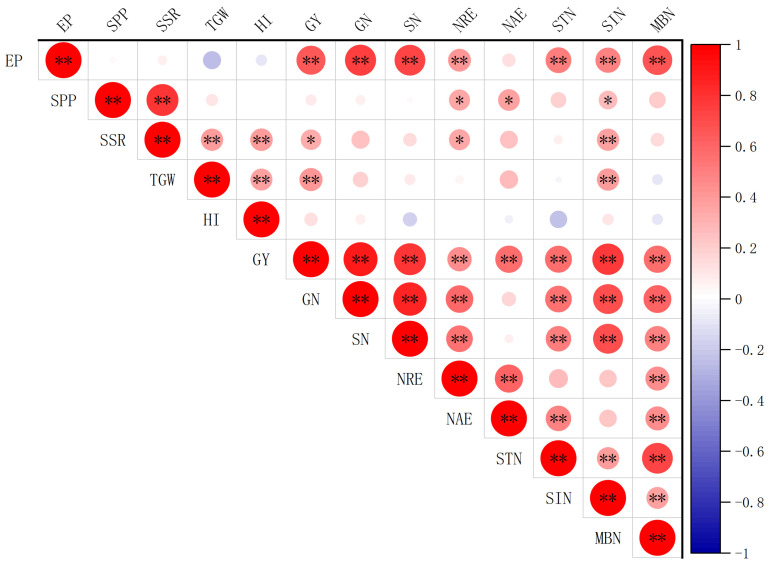
Pearson correlation matrix among yield components, nitrogen uptake parameters, nitrogen use efficiency indices, and soil nitrogen pools.* and ** indicate significance at *p* < 0.05 and *p* < 0.01, respectively. The intensity of color increases with the magnitude of correlation coefficients. EP, effective panicles; SPP, spikelets per panicle; SSR, seed setting rate; TGW, thousand-grain weight; HI, harvest index; GY, grain yield; GN, grain N uptake; SN, straw N uptake; NRE, nitrogen recovery efficiency; NAE, nitrogen agronomic efficiency; STN, soil total nitrogen; SIN, soil inorganic nitrogen; MBN, soil microbial biomass nitrogen.

Based on the results of the correlation analysis (Figure 8), grain yield was highly significantly positively correlated (*p* < 0.01) with effective panicle number, thousand-grain weight, grain N uptake, straw N uptake, apparent N recovery efficiency, agronomic N efficiency, soil total N, soil inorganic N, and soil microbial biomass N, and significantly positively correlated (*p* < 0.05) with seed setting rate. Apparent N recovery efficiency was highly significantly positively correlated (*p* < 0.01) with effective panicle number, grain yield, grain N uptake, straw N uptake, agronomic N efficiency, and soil microbial biomass N, and significantly positively correlated (*p* < 0.05) with spikelet number per panicle and seed setting rate. Agronomic N efficiency was highly significantly positively correlated (*p* < 0.01) with grain yield, apparent N recovery efficiency, soil total N, and soil microbial biomass N and significantly positively correlated (*p* < 0.05) with spikelet number per panicle. Among the yield components, effective panicle number was the primary factor affecting grain yield.

### 2.7. Economic Benefit

As shown in Table 3, the fertilizer costs of CRU1 and CRU2 were 258 and 179 Yuan ha^−1^ higher than those of FP, respectively, while their net economic benefits were 24.5% and 18.9% higher than that of FP. This was because the labor cost for the one-time basal fertilization was only 1200 Yuan ha^−1^, saving 2400 Yuan ha^−1^ compared with the three split applications in FP. In addition, CRU2 had the highest output value, which was 2229 Yuan ha^−1^ (9.6%) higher than that of FP. FPM had the lowest net economic benefit among all N application treatments, which was 6.4% lower than that of FP, mainly due to its high fertilizer cost and multiple fertilization applications. FPS had a net economic benefit of 19,641.20 Yuan ha^−1^, which was 8.3% higher than that of FP.

Overall, although the unit price of slow-release fertilizer was higher, it significantly improved net economic benefits by reducing labor costs and increasing yield. CRU2 showed the best input–output ratio, with a net benefit nearly one-quarter higher than that of FP.

Although the fertilizer costs of CRU1 and CRU2 were higher than that of FP, their net economic benefits were both higher than that of FP due to the saving in labor cost (reduced from three fertilizations to one). CRU2 was the most economical model, with the highest net economic benefit of 22,580 Yuan ha^−1^, which was 24.5% higher than that of FP and 35.6% higher than that of CK. Although the unit price of slow-release nitrogen fertilizer was 45% higher than that of common urea, CRU2 achieved a higher net benefit by reducing the number of fertilization events and optimizing the blending ratio, indicating that the higher price of slow-release fertilizer could be offset by the savings in labor costs and the gains in yield.

## 3. Discussion

### 3.1. CRU2 Enhances Grain Yield Through Synchronization of Nitrogen Supply with Crop Demand

The 5:3:2 blending ratio was designed with reference to the fertilization ratio of conventional split application (FP), i.e., basal:tillering:panicle fertilizer = 50%:30%:20%. This aimed to achieve the goal of “one-time basal application with staged nitrogen supply” through the combination of slow-release fertilizers with different release durations. The different nutrient management regimes significantly affected rice yield, with the CRU2 treatment showing the most pronounced yield-enhancing effect over the 3-year field experiment, with an average yield improvement of 9.6% compared with conventional fertilization (FP). This result is consistent with the design concept of the “fast–steady–slow” N supply pattern for blended slow-release fertilizers [18], confirming that the rational combination of N fertilizers with different release periods can achieve efficient synchronization between N supply and the stage-specific N demand of rice.

Rice N uptake and use exhibit distinct stage-specific characteristics: the tillering stage is the first peak period of N uptake, where adequate N supply promotes early growth and effective tiller formation. The jointing to booting stage has a steadily increasing N demand to support panicle differentiation, whereas the grain-filling stage still requires a certain amount of N to ensure grain plumpness [19]. The N ratio design of CRU2 fully matched this N demand pattern: the application of 50% conventional urea met the N demand during the tillering stage after transplanting, promoting rapid tiller emergence. The application of 30% 40-day slow-release urea corresponded to the N uptake peak from peak tillering to jointing, ensuring tiller survival and panicle differentiation. The application of 20% 90-day slow-release urea matched the N demand from panicle differentiation to heading and grain filling, ensuring seed setting rate and grain weight. This N supply pattern—“readily available N promotes tillering in the early stage, stable N supply ensures panicle number in the middle stage, slow-release N promotes grain filling in the later stage”—achieved dynamic synchronization between N release and rice uptake [20].

According to previous studies, the degree of matching between the N release characteristics of slow/controlled-release fertilizers and rice N demand directly affects yield and yield components [21,22]. Zhang et al. found that a single basal application of controlled-release fertilizer better matched the nutrient uptake patterns of rice, ensuring tiller formation at the early stage while maintaining stable photosynthetic production after heading, thereby achieving a higher panicle number, spikelet number per panicle, and thousand-grain weight [23]. In this study, the yield increase in CRU2 treatment was mainly a result of the significant increase in effective panicle number, with a 3-year average of 341.6 × 10^4^ panicles ha^−1^, which was 10.2% higher than that in FP (*p* < 0.05); in contrast, spikelet number per panicle and seed setting rate, although higher than those in the other fertilization treatments, did not reach significant levels. This difference may be related to the release characteristics of the combination of conventional urea and different slow/controlled-release fertilizers. Previous studies mostly used slow-release fertilizers with a single release period such as 60 or 90 days, ensuring sufficient N supply in the later stage, which is beneficial for increasing spikelet number per panicle and thousand-grain weight [24]. In contrast, we used a “fast-acting + medium-acting + long-acting” composite ratio for CRU2, with the N matching advantage mainly reflected in the precise supply of N during the early tillering stage. Matching N uptake during the tillering stage increases the effective panicle number. By strengthening early-stage tillering N supply and meeting later-stage N requirements, CRU2 effectively promoted the formation of effective tillers and panicles while stabilizing thousand-grain weight and spikelet number per panicle, which is the key mechanism for its yield advantage.

During the three-year rice growing seasons, mean temperatures differed markedly. In 2022, the mean temperature was 25.5 °C, which was 0.3 °C below the long-term average of 25.8 °C. In 2023, the mean temperature was 26.6 °C, which was 0.8 °C above the long-term average. In 2024, the mean temperature was 24.9 °C, which was 0.9 °C below the long-term average. These temperature differences were consistent with the observed yield performance. The significant year-to-year variations in traits such as thousand-grain weight and seed setting rate were mainly attributed to the differences in mean temperature during the growing season.

Thus, when the release characteristics of slow/controlled-release fertilizers match rice N demand in different ways, the responses of yield components also differ. When the matching advantage is reflected in the early stage, the effective panicle number increases more significantly. In contrast, when the matching advantage is reflected in the middle and later stages, it is more conducive to increases in spikelet number per panicle and thousand-grain weight.

### 3.2. Nitrogen Synchrony Under CRU2 Enhances N Uptake Efficiency and Soil Nitrogen Fertility

CRU2 treatment not only achieved yield advantages but also higher N uptake and use efficiency. The 3-year average grain N uptake reached 125.46 kg ha^−1^, which was 12.1% higher than that achieved via FP. Straw N uptake reached 72.84 kg ha^−1^, which was 21.4% higher than that in FP. The apparent N recovery efficiency reached 58.52%, which was 15.98% higher than that in FP. The agronomic N efficiency reached 16.55 kg kg^−1^, which was 5.40 kg kg^−1^ higher than that in FP. The partial factor productivity of nitrogen (PFPN) reached 61.94 kg kg^−1^, which was 5.40 kg kg^−1^ higher than that of FP. The N matching advantage of CRU2 was reflected in two aspects.

First, the combination of N fertilizers with different release periods matched the stage-specific demands of rice, increasing tiller number, enhancing N uptake, and reducing ineffective N losses. Apparent N balance analysis showed that CRU2 had a 3-year average apparent N balance of −33.0 kg ha^−1^, the largest negative value among fertilized treatments except that of the unfertilized control, indicating that crop N uptake significantly exceeded N input, reflecting the highest N use efficiency. The combination of 40-day and 90-day slow-release urea in CRU2 ensured N supply for the current crop while reducing rapid N release and losses through the slow-release effect, allowing more N to be absorbed and used by the crop [25].

Second, at the soil N level, CRU2 significantly increased the soil inorganic N content. The 3-year average data show that the soil inorganic N content under CRU2 was 27.9% higher than that under FP, with significant differences. Consistent with the findings of Quan et al., the combined application of controlled-release N fertilizer and urea can increase the soil-available N content and microbial activity, promoting N transformation and crop uptake [26]. Whilst achieving a high crop N uptake, CRU2 maintained a significantly higher soil inorganic N content than conventional fertilization. On the one hand, the sustained supply of slow-release N fertilizer maintained stable available N concentrations in the soil, ensuring a high inorganic N content at the sampling time; on the other hand, continuous N supply stimulated microbial activity, significantly increasing microbial biomass N as an active organic N pool, whilst the rapid turnover of microorganisms promoted the mineralization of native soil organic N, providing an additional N source beyond the current season’s fertilizer input [27]. In this study, soil nitrate N content was generally low across all treatments, with three-year average values ranging from 2.03 to 2.91 mg kg^−1^, accounting for a relatively small proportion of total inorganic N. Moreover, the amount of residual nitrate N in the soil after rice harvest was limited. Therefore, the risk of nitrate N leaching to water bodies in the short term was low, and this issue was not a primary concern of this study.

The FPS and FPM treatments performed well in terms of soil total N and microbial biomass N. FPS treatment had a 3-year average total N content 15.2% higher than that of FP, whereas FPM had a microbial biomass N level 30.8% higher than that of FP, which was significantly different. In the FPM treatment, part of the N was derived from bio-organic manure. In the FPS treatment, the straw-borne N exceeded the 165 kg N ha^−1^ limit, which likely accounts for the higher soil total N after harvest. Nevertheless, straw decomposition consumed some N, presumably at the expense of rice N nutrition.

This indicates that straw return and bio-organic manure substitution have unique advantages in building soil organic N pools and stimulating microbial activity, but their N supply matching with rice N demand is inferior to that of CRU2 treatment, resulting in limited increases in yield and N use efficiency. This result suggests that the different nutrient management regimes have their intrinsic advantages. In practical production, optimized combinations can be selected based on target goals: if high yield and high efficiency are the primary goals, CRU2 is the preferred choice; if soil fertility building is the focus, integrated models combining FPS or FPM with efficient N fertilizers can be considered.

### 3.3. Apparent Nitrogen Balance, Cost Accounting, and Sustainability Analysis of CRU2

In this study, the apparent N balance was calculated simply as the difference between fertilizer N input and aboveground crop N uptake, without accounting for N losses via ammonia volatilization, nitrate leaching, or denitrification. The main reason for this simplification is that the pathways and emission factors of N loss vary considerably among different fertilization treatments, making it difficult to accurately quantify the N loss flux for each treatment [28]. Therefore, a simplified apparent N balance approach was adopted.

In the apparent N balance calculation, the nitrogen input for both the FPM and FPS treatments included the nitrogen derived from their respective organic sources (bio-organic manure and straw return). However, in the fertilizer application rates presented in Table 4, only the nitrogen derived from bio-organic manure in the FPM treatment was included, while the nitrogen contributed by straw return in the FPS treatment was not accounted for. This is mainly because straw has a high C/N ratio, and during straw decomposition, microorganisms assimilate a large amount of inorganic N, resulting in a low release rate of straw N in the current season. Consequently, most of the straw N remains in the soil as organic N and is not readily available for direct uptake by rice [29,30]. When organic N sources were included, FPS showed a N surplus, which is consistent with the soil total N results showing that FPS had a significantly higher total N content than FP.

In agricultural practice, fertilization decisions are influenced not only by yield and nitrogen use efficiency but also directly by fertilizer costs and labor input. The fertilizer costs for CRU1 and CRU2 were 1950 and 1771 Yuan ha^−1^, respectively, which were 358 and 179 Yuan ha^−1^ higher than those of conventional fertilization (FP). This increase was mainly due to the 45% higher unit price of slow-release nitrogen fertilizer compared with common urea. However, both CRU1 and CRU2 were applied as a single basal dressing, resulting in a labor cost of only 1200 Yuan ha^−1^, which was 2400 Yuan ha^−1^ lower than that of FP. Therefore, although the fertilizer costs of the slow-release treatments were higher, the savings in labor costs more than offset this increase. The net economic benefits of CRU1 and CRU2 were 21,572 and 22,580 Yuan ha^−1^, respectively, representing increases of 19.0% and 24.5% compared with FP. Comprehensive economic analysis indicates that CRU2 achieved the best input–output ratio. Although the unit price of slow-release nitrogen fertilizer was higher, the reduction in fertilization frequency saved labor costs, and the higher yield output maximized net returns. Therefore, from the perspective of farmer decision-making, CRU2 is the recommended fertilization model for achieving high yield, high efficiency, and economic profitability.

Although CRU2 produced the highest grain yield and the most favorable N use efficiencies (NRE, NAE, and N uptake), it also exhibited the largest negative apparent N balance, implying substantial N uptake from mineralized soil organic matter. Consequently, prolonged application of CRU2 may adversely affect soil properties, particularly soil organic matter content. In contrast, FPM and FPS, despite their lower yields during the experimental period, contributed to soil improvement, whereas CRU2 appeared to intensively exploit soil nutrient reserves. Over the long term, the yield advantage may therefore shift toward the latter two treatments. However, the absolute value of the negative nitrogen balance under CRU2 was relatively small, and the remaining nitrogen retained in the soil had a limited effect on soil nitrogen content in the short term. Moreover, the soil total nitrogen content did not show a declining trend over the three years. In the long term, the sustainability of soil nitrogen could be maintained by incorporating straw return.

## 4. Materials and Methods

### 4.1. Site Description

The long-term field experiment was established in 2019, and the present study was conducted from June 2022 to October 2024 in Jilongshan Village, Xibu Town, He County, Ma’anshan City, Anhui Province, China (31°46′32″ N, 118°12′58″ E). The climate is a northern subtropical humid monsoon climate, with a mean annual temperature of 15.7 °C and an average annual precipitation of 1000 mm. The soil at the experimental site is yellow-brown soil, without distinct restrictive layers within the profile. The basic physicochemical properties of the topsoil (0–20 cm) prior to the experiment were as follows: organic matter content, 19.3 g kg^−1^; pH (H_2_O), 6.12; total nitrogen (N), 1.25 g kg^−1^; available phosphorus, 33.8 mg kg^−1^; and available potassium, 140.3 mg kg^−1^.

### 4.2. Experimental Design and Treatments

We employed a randomized complete block design with three replications. Each plot measured 60 m^2^ (6 × 10 m). Six distinct nutrient management treatments were established, as detailed below.

Control (CK): No N fertilizer was applied. Basal application of all P and K fertilizers; no topdressing.

Conventional Farmer Practice (FP): Nitrogen was applied as urea at 165 kg N ha^−1^, with 50% basally incorporated, 30% topdressed at the tillering stage, and 20% topdressed at the booting stage.

Single Basal Application of 60-day SRU (CRU1): Nitrogen was applied at 1665 kg N ha^−1^ solely as a one-time basal dressing of 60-day slow-release urea (SRU), with no subsequent topdressing.

Blended Slow-Release Urea (CRU2): Nitrogen was applied at 165 kg N ha^−1^ as a one-time basal dressing, consisting of a blend of granular urea, 40-day SRU, and 90-day SRU in a ratio of 5:3:2 (on a N basis). No topdressing was applied.

Bio-organic Manure Substitution (FPM): Nitrogen was applied at a total rate of 165 kg N ha^−1^, with 34.5% of the total N supplied by bio-organic manure. The remaining N was applied as urea. The full dose of P and K fertilizers, the bio-organic manure, and 50% of the urea-N were applied basally. The remaining urea was topdressed, with 30% at tillering and 20% at booting.

Straw Return with Conventional Practice (FPS): Nitrogen was applied as urea at 165 kg N ha^−1^, following the same splitting ratio as FP (50% basal, 30% at tillering, and 20% at booting). In addition, all aboveground rice straw from the previous season was chopped and incorporated into the soil shortly before transplanting.

The specific N application rates for each treatment are presented in Table 4. The study was conducted under the rice–wheat rotation system. The rice cultivar used was “*Changliangyou*”, and seedlings were transplanted at a density of 1.98 × 10^5^ hills ha^−1^. Agronomic management practices, including irrigation and pest control, followed the standard protocols for high-yielding rice used in the study region. Plots were separated by cement-covered ridges to prevent water and nutrient cross-contamination, and each plot was equipped with an independent irrigation and drainage system.

Urea (46% N), triple superphosphate (42% P_2_O_5_), and potassium chloride (60% K_2_O) were used as conventional fertilizers. The slow-release nitrogen fertilizer (44.5% N) was supplied by Anhui Moith Agricultural Technology Co., Ltd. (Anhui, China), with polyurethane-coated urea as its core component. Yuan et al. determined the release characteristics of 40-day and 90-day slow-release nitrogen fertilizers under paddy field conditions and in water at 25 °C, respectively, showing that both fertilizers exhibited stable release rates in the two environments, following a cubic binomial curve. Specifically, the nitrogen release rates of the 40-day slow-release fertilizer in the paddy field and in water at 25 °C were 89.7% and 86.7%, respectively, while those of the 90-day slow-release fertilizer were 91.2% and 87.4%, respectively [31].

Wheat straw was returned to the field after harvest at a rate of 4500 kg ha^−1^, with a C/N ratio of 72 and a nitrogen content of 0.62%. Bio-organic manure was applied at 3000 kg ha^−1^, with a total nitrogen content of 1.9% and a C/N ratio of 12. The nitrogen input from bio-organic manure included both mineral N and organic N, Table 5.

### 4.3. Sampling and Measurements

#### 4.3.1. Plant Sampling and Yield Determination

At physiological maturity, grain yield was determined by manually harvesting a central area of 30 m^2^ from each plot. For yield component analysis, five representative hills with uniform growth were randomly selected from each plot. Plants were separated into aboveground straw (culm, leaf sheaths, and leaf blades) and panicles. All plant samples were oven-dried at 105 °C for 30 min to inactivate enzymes, then further dried at 75 °C to constant weight to determine dry matter accumulation.

#### 4.3.2. Soil Sampling and Analysis

Immediately after the rice harvest, soil samples were collected from the 0–20 cm plow layer using a stainless-steel auger (5 cm diameter). Five cores were randomly taken from each plot and composited into a single sample. After the removal of roots and debris, the composite sample was passed through a 5 mm sieve to ensure homogeneity. A subsample of fresh soil was immediately stored in a cooler at 4 °C for the determination of soil microbial biomass N (MBN), ammonium N (NH_4_^+^-N), and nitrate N (NO_3_^−^-N). The remaining soil was air-dried naturally, ground, and passed through a 0.15 mm sieve for the analysis of total N (TN).

Soil Total Nitrogen (TN): Total nitrogen was determined by the Kjeldahl method. An air-dried soil sample of 0.2000 g was weighed and digested with concentrated H_2_SO_4_ and a catalyst, followed by distillation and titration using Kjeldahl apparatus [32]. The Kjeldahl apparatus was purchased from Jinan Hanon Instruments Co., Ltd. (Jinan, Shandong, China).

Soil Nitrate Nitrogen (NO_3_^−^-N)/Soil Ammonium Nitrogen (NH_4_^+^-N): Soil ammonium N and nitrate N were extracted with 2 mol·L^−1^ KCl solution by shaking at 180 r·min^−1^ for 1 h, and the extracts were analyzed using an AA3 continuous flow analyzer [33]. The AA3 continuous flow analyzer, purchased from SEAL Analytical (Shanghai) Co., Ltd. (manufactured in Germany).

Soil Microbial Biomass Nitrogen (MBN) was determined using the chloroform fumigation–K_2_SO_4_ extraction method. Two portions of fresh soil (each equivalent to 10 g of dry soil) were taken. One portion was fumigated with chloroform in a vacuum desiccator for 24 h, while the other portion was not fumigated and served as the control. After fumigation, both soil portions were extracted with 0.5 mol L^−1^ K_2_SO_4_ solution at a soil-to-solution ratio of 1:4 by shaking for 30 min. The extracts were filtered, and the total nitrogen content in the extracts was analyzed using a total organic carbon/total nitrogen (TOC/TN) analyzer. MBN was calculated as follows [34]:

MBN = (N extracted from fumigated soil − N extracted from non-fumigated soil)/0.54

#### 4.3.3. Plant Nitrogen Analysis

At rice maturity, five representative hills with uniform growth were randomly selected from each plot. The plants were separated into grains and straw. The plant samples were placed in an oven at 105 °C for 30 min to deactivate enzymes. Oven-dried straw and grain samples were ground to a fine powder using a stainless-steel grinder. A subsample of approximately 0.2 g was weighed and digested with H_2_SO_4_-H_2_O_2_. The digest was cooled, diluted 10-fold with deionized water, and thoroughly mixed. The total N concentration in the digest was determined using an automated Kjeldahl analyzer. Plant total nitrogen content was determined using the same Kjeldahl apparatus as that used for soil total nitrogen, purchased from Jinan Hanon Instruments Co., Ltd. (Jinan, Shandong, China).

### 4.4. Data Analysis

The following parameters were calculated for each treatment:

Nitrogen apparent recovery efficiency (*NRE*, %) was determined using the following formula [35]:

$$NRE(\%)=\frac{U_{N}-U_{0}}{F_{N}}\times 100,$$

where *U_N_* and *U_0_* are the total aboveground N uptake (kg ha^−1^) in the N-fertilized plot and the unfertilized control (CK) plot, respectively, and *F_N_* is the rate of N fertilizer applied (kg ha^−1^).

Nitrogen agronomic efficiency (*NAE*, kg kg^−1^) was determined as follows [35]:

$$NAE({\mathrm{Kg}\;\mathrm{kg}}^{-1})=\frac{Y_{N}-Y_{0}}{F_{N}},$$

where *Y_N_* and *Y*_0_ are the grain yields (kg ha^−1^) in the N-fertilized plot and the unfertilized control (CK) plot, respectively.

*Apparent N balance* (kg N ha^−1^): A simplified N budget was calculated to indicate the potential surplus or deficit [36], using the following equation:

$$ApparentNBalance=F_{N}-U_{N},$$

where a positive value indicates a potential N surplus (e.g., N loss or residual soil N) and a negative value indicates N loss from the soil.

Partial factor productivity of nitrogen (*PFPN*, kg kg^−1^):

$$PFPN({\mathrm{Kg}\;\mathrm{kg}}^{-1})=\frac{GY}{N}$$

where *GY* is the grain yield in the fertilized plot (kg ha^−1^) and *N* is the nitrogen application rate (kg ha^−1^). A higher PFPN value indicates greater productivity per unit of nitrogen fertilizer input.

Net economic benefit = Output value − Labor cost − Fertilizer cost

### 4.5. Statistical Analysis

Data processing and preliminary calculations were performed using Microsoft Excel 2021. All graphical representations were created with OriginPro 2024. Statistical analyses were conducted using IBM SPSS Statistics version 24.0. One-way analysis of variance (ANOVA) was employed to test the significance of treatment effects on all measured variables. When a significant F-test (*p* < 0.05) was obtained, treatment means were compared using the least significant difference (LSD) test at the 5% probability level.

## 5. Conclusions

This 3-year field experiment demonstrated that the CRU2 treatment (single basal application of 50% conventional urea combined with 30% 40-day slow-release urea and 20% 90-day slow-release urea) achieved efficient synchronization between N supply and the dynamic N demand of rice throughout its growth stages (tillering in the early stage, jointing in the middle stage, and grain filling in the later stage). It significantly increased effective panicle number and grain yield while improving N use efficiency, making it the optimal nutrient management strategy for rice cultivation in the Jianghuai region.

## Figures and Tables

**Table 1:** Yield components of rice under different nutrient management regimes (2022–2024).

Year	Treatment	Spikelets per Panicle	Seed Setting Rate (%)	1000-Grain Weight (g)	Effective Panicles (×10^4^ ha^−1^)	Harvest Index (%)
2022	CK	151.10 ± 4.25 b	84.00 ± 1.11 b	21.93 ± 0.12 a	276.43 ± 5.74 c	52.74 ± 2.39 a
	FP	166.93 ± 4.26 a	85.00 ± 0.92 ab	21.62 ± 0.18 a	321.63 ± 3.69 b	53.53 ± 2.67 a
	CRU1	166.13 ± 5.68 a	85.70 ± 1.25 ab	21.97 ± 0.15 a	338.27 ± 26.34 ab	53.12 ± 2.21 a
	CRU2	170.47 ± 5.63 a	86.90 ± 1.35 a	21.62 ± 0.55 a	359.27 ± 6.67 a	54.57 ± 3.16 a
	FPM	165.47 ± 7.68 a	85.00 ± 1.41 ab	22.08 ± 0.08 a	334.90 ± 20.52 ab	52.82 ± 2.08 a
	FPS	165.73 ± 1.75 a	85.20 ± 0.89 ab	22.02 ± 0.15 a	337.70 ± 19.88 ab	52.97 ± 0.33 a
2023	CK	156.30 ± 3.01 b	84.00 ± 1.41 b	23.43 ± 0.41 a	248.00 ± 9.82 c	53.80 ± 1.86 a
	FP	168.87 ± 7.72 a	85.70 ± 1.45 ab	23.55 ± 0.29 a	297.60 ± 10.41 b	55.22 ± 1.08 a
	CRU1	171.70 ± 3.60 a	86.00 ± 0.92 ab	23.66 ± 0.26 a	306.47 ± 10.85 ab	52.27 ± 2.37 a
	CRU2	174.33 ± 2.90 a	87.20 ± 1.11 a	23.47 ± 0.33 a	321.17 ± 5.69 a	53.75 ± 2.45 a
	FPM	169.07 ± 9.56 a	85.40 ± 1.51 ab	23.48 ± 0.15 a	301.13 ± 14.15 ab	51.55 ± 1.87 a
	FPS	170.97 ± 6.01 a	85.90 ± 1.05 ab	23.17 ± 0.29 a	303.60 ± 12.19 ab	51.65 ± 1.56 a
2024	CK	151.67 ± 6.55 b	83.00 ± 1.41 a	20.33 ± 0.64 a	264.83 ± 10.79 c	51.70 ± 1.73 a
	FP	168.87 ± 9.72 a	84.30 ± 1.85 a	20.87 ± 0.71 a	310.17 ± 13.90 b	49.88 ± 1.26 ab
	CRU1	172.17 ± 6.21 a	84.70 ± 1.45 a	22.40 ± 0.56 a	332.23 ± 14.98 ab	50.03 ± 1.27 ab
	CRU2	174.57 ± 8.95 a	85.30 ± 1.55 a	21.03 ± 0.83 a	344.40 ± 7.40 a	50.26 ± 1.62 ab
	FPM	168.80 ± 7.75 a	84.10 ± 1.55 a	21.02 ± 0.40 a	319.57 ± 15.11 ab	50.80 ± 1.56 ab
	FPS	171.03 ± 6.99 a	84.40 ± 1.55 a	20.65 ± 0.70 a	330.03 ± 16.32 ab	48.38 ± 0.51 b
Source of variation						
Year (Y)		ns	**	**	**	**
Treatment (T)		**	**	ns	**	ns
Y × T		ns	ns	ns	**	ns

Values are means ± standard deviation (*n* = 3). Within a column and year, values followed by different lowercase letters are significantly different at *p* < 0.05. **, *p* < 0.01; ns, not significant.

**Table 2:** Apparent nitrogen balance under different nutrient management regimes (2022–2024).

Treatment		2022		2023		2024	
	N Input (kg ha^−1^)	N Output (kg ha^−1^)	N Balance (kg ha^−1^)	N Output (kg ha^−1^)	N Balance (kg ha^−1^)	N Output (kg ha^−1^)	N Balance (kg ha^−1^)
CK	0	100 ± 5 d	−100 ± 5 d	106 ± 10 c	−106 ± 10 c	99 ± 9 d	−99 ± 9 d
FP	165	174 ± 12 bc	−9 ± 12 bc	181 ± 11 b	−16 ± 11 b	161 ± 9 bc	4 ± 9 bc
CRU1	165	176 ± 6 bc	−11 ± 6 bc	181 ± 4 b	−16 ± 4 b	172 ± 7 bc	−7 ± 7 bc
CRU2	165	195 ± 2 a	−30 ± 2 a	204 ± 6 a	−39 ± 6 a	195 ± 10 a	−30 ± 10 a
FPM	165	159 ± 6 c	6 ± 6 c	168 ± 7 b	−3 ± 7 b	158 ± 17 c	7 ± 17 c
FPS	193	191 ± 18 ab	2 ± 18 ab	181 ± 4 b	12 ± 4 b	177 ± 5 b	16 ± 5 b

Values are means ± standard deviation *(n* = 3). Within a column, values followed by different lowercase letters are significantly different at *p* < 0.05. N balance was calculated as N input minus crop N uptake; negative values indicate net soil N mining or efficient utilization of applied N, while positive values indicate surplus N potentially lost to the environment. Treatment abbreviations are defined in Figure 1.

**Table 3:** Effects of different nutrient management regimes on the economic benefits of each treatment.

Treatment	Output Value	Labor Cost	Fertilizer Cost	Net Economic Benefit
	(Yuan ha^−1^)	(Yuan ha^−1^)	(Yuan ha^−1^)	(Yuan ha^−1^)
CK	18,725	1200	875	16,650
FP	23,322	3600	1592	18,130
CRU1	24,722	1200	1950	21,572
CRU2	25,552	1200	1771	22,580
FPM	24,013	3600	3445	16,968
FPS	24,834	3600	1592	19,641

During the 2022–2024 experimental period, the average prices of urea, potassium fertilizer, phosphate fertilizer, slow-release nitrogen fertilizer, and bio-organic manure were 2, 3, 3.5, 2.9, and 0.7 Yuan kg^−1^, respectively, and the average selling price of rice was 2.5 Yuan kg^−1^. This study focused on the economic effects of nitrogen fertilizer types and their blending ratios; therefore, other production costs were not included, and the resulting net economic benefit represents a partial net economic benefit. Output values were calculated based on the three-year average yield. Treatment abbreviations are defined in Figure 1.

**Table 4:** Fertilizer application rates under different treatments (kg ha^−1^).

Treatment		Fertilizer Application Rates										
	Basal Fertilizer							Topdressing		Total Application		
	Chemical N	Chemical P	Chemical K	Organic Material	40-day SRU	90-day SRU	60-day SRU	Tillering	Booting	N	P_2_O_5_	K_2_O
CK	0	60	75	0	0	0	0	0	0	0	60	75
FP	82.5	60	75	0	0	0	0	49.5	33	165	60	75
CRU1	0	60	75	0	0	0	165	0	0	165	60	75
CRU2	82.5	60	75	0	49.5	33	0	0	0	165	60	75
FPM	54	60	75	3000	0	0	0	32.4	21.6	165	120	105
FPS	82.5	60	75	4500	0	0	0	49.5	33	165	60	75

SRU, slow-release urea. All P and K fertilizers were applied as basal. The nitrogen application rate was 165 kg N ha^−1^ for all fertilized treatments. In this study, the nitrogen, phosphorus, and potassium from bio-organic manure were included in the nutrient input calculation, whereas those from straw were not included.

**Table 5:** Key growth stages and field management practices of rice during 2022–2024.

2022		2023		2024	
Date	Management Practices	Date	Management Practices	Date	Management Practices
27 May	Sowing	28 May	Sowing	27 May	Sowing
13 June	Basal fertilizer application	14 June	Basal fertilizer application	12 June	Basal fertilizer application
14 June	Transplanting	15 June	Transplanting	13 June	Transplanting
30 June	Tillering fertilizer application	1 July	Tillering fertilizer application	2 July	Tillering fertilizer application
25 July	Mid-season drainage	20 July	Mid-season drainage	23 July	Mid-season drainage
10 August	Panicle fertilizer application	7 August	Panicle fertilizer application	9 August	Panicle fertilizer application
18 October	Maturity—rice harvest	22 October	Maturity—rice harvest	16 October	Maturity—rice harvest

## Data Availability

The original contributions presented in the study are included in the article. Further inquiries can be directed to the corresponding authors.
